# Terson Syndrome Diagnosed by Ocular Point of Care Ultrasound on the Medical Floor

**DOI:** 10.24908/pocus.v9i1.16660

**Published:** 2024-04-22

**Authors:** Mark Johnson

**Affiliations:** 1 Department of Long-Term Unscheduled Care, Harrogate and District Foundation Trust Harrogate, Yorkshire UK

**Keywords:** POCUS, Terson Syndrome, Viterous Haemorrhage, Ocular Ultrasound, Internal Medicine, Subarachnoid Haemorrhage

## Abstract

In acute care environments, accurately assessing complications of intracranial pathology can be challenging. Ocular complications in acute intracranial disease are not consistently evaluated despite their high morbidity. We report on a case of monocular diplopia in a 63-year-old man with subacute traumatic brain injury with localized subarachnoid hemorrhage. Ocular point of care ultrasound (POCUS) identified features of vitreous hemorrhage in one globe, leading to a diagnosis of Terson syndrome and a timely referral to ophthalmology. This finding was made on the medical floor days after the initial presentation during rehabilitation when ophthalmoscopy was not possible, and vitreous hemorrhage had not been identified on presentation. Terson syndrome is a seldom discussed but important complication of intracranial hemorrhage generally associated with poor patient outcomes. Ocular POCUS can provide a useful alternative in assessing ocular complications of acute intracranial disease on the medical floor, particularly when the practicalities of performing ophthalmoscopy are challenged.

## Introduction

Managing intracranial pathology and its associated complications is a common challenge for acute care providers. Intracranial pathology can present with a myriad of signs and symptoms which may be challenging to elicit through examination alone 1. As well, many of these complications gain increasing morbidity and mortality in our ageing population 1. Regardless of traumatic or non-traumatic mechanisms, ocular manifestations of intracranial pathology can range from clinically apparent to subtle, with most presentations having potentially sight-threatening outcomes 2. As a result, ophthalmoscopy is often advised to complete the evaluation of a patient with suspected intracranial pathology. Such patients can be difficult to interview or examine for visual disturbance – particularly in cases of altered mental status commonly associated with intracranial pathology – complicating accurate clinical evaluation. 

It can be challenging to assess the visual system in acute care settings. The challenges include a need to monitor pupillary responses in acute intracranial injury precluding mydriasis, the inability to darken specific environments, and reduced access to ophthalmoscopy equipment in certain locations, such as resuscitation areas 3. As a result of these factors, 59% of ocular assessments in the Emergency Department are deemed inadequate 4. These factors have led to a compounding cycle of increasing reliance on specialist assessment, reduced non-specialist competence, and reduced engagement in medical education 5.

In an era of point of care ultrasound (POCUS) expansion, increasing access to linear sonographic devices and decline of non-specialist ophthalmology, it is worth recalling the benefits of ocular POCUS. Here we demonstrate a case where ocular POCUS identified ocular complications of traumatic intracranial pathology, specifically traumatic cerebral contusion with localized subarachnoid hemorrhage. This complication had not been identified during the patient's initial assessment despite thorough review from numerous clinicians. The diagnosis of this complication in the care of acute intracranial pathology patients can reduce morbidity through expediting access to specialist care to preserve sight as well as provide useful prognostic information at the bedside. 

## Case Report

A 63-year-old man presented to the Emergency Department suffering with irretractable nausea and vomiting with concomitant severe headache following a fall at home three days prior. The fall occurred from a standing height and was associated with minor abrasions to the forehead. The patient’s family highlighted that the patient had seemed increasingly confused in the last 24 hours; this had initially been managed conservatively as a concussion symptom. 

**Figure 1:**
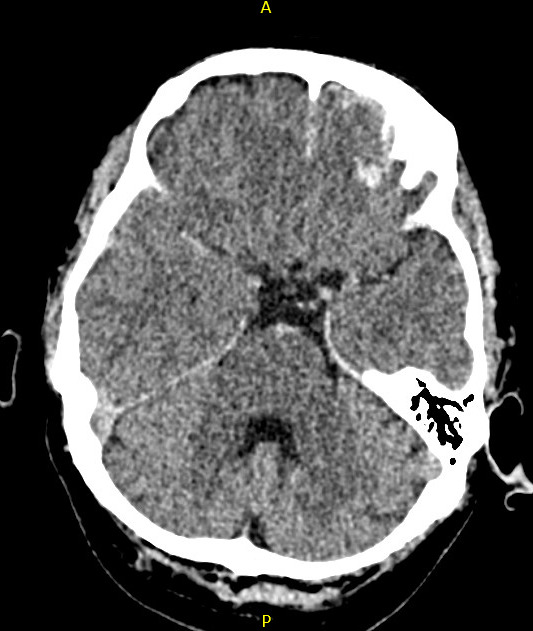
Small left frontal cerebral contusion with localized traumatic subarachnoid hemorrhage.

On examination, the patient was neurologically intact with mild confusion, reflected in an Abbreviated Mental Test Score (AMTS) of 8 out of 10. The patient reported no visual disturbance, and no further systemic pathology was identified. The patient was referred for computed tomography (CT) of the head which revealed a predominantly left-sided cerebral contusion without fracture to the skull with localized traumatic subarachnoid hemorrhage (Figure 1). This result was discussed with the regional neurosurgical center who elected for local admission and observation. No evidence of ocular pathology was present on the head CT. The patient was admitted to a medical bed for observation and consultation under neurology. Four days following admission, with symptom management and close observation, the patient became increasingly orientated. During a rehabilitative physiotherapy assessment, visual field difficulties were noted and the patient reporting that vision from his left eye was altered. 

On examination, the patient demonstrated normal ocular movements and no external ocular injuries were appreciated. The patient described monocular diplopia on isolation of the left eye visual field. Under formal evaluation of his visual acuity using a Snellen chart, the patient had an intact right eye visual acuity (6/6), with a reduction in left eye visual acuity (3/6). The patient could not undergo ophthalmoscopy due to an unfavorable bright ward environment, a lack of mydriatic medication, and difficulty following instructions. As a result, the decision was made to proceed to ocular POCUS in the inpatient ward setting.

### POCUS Assessment

**Figure 2:**
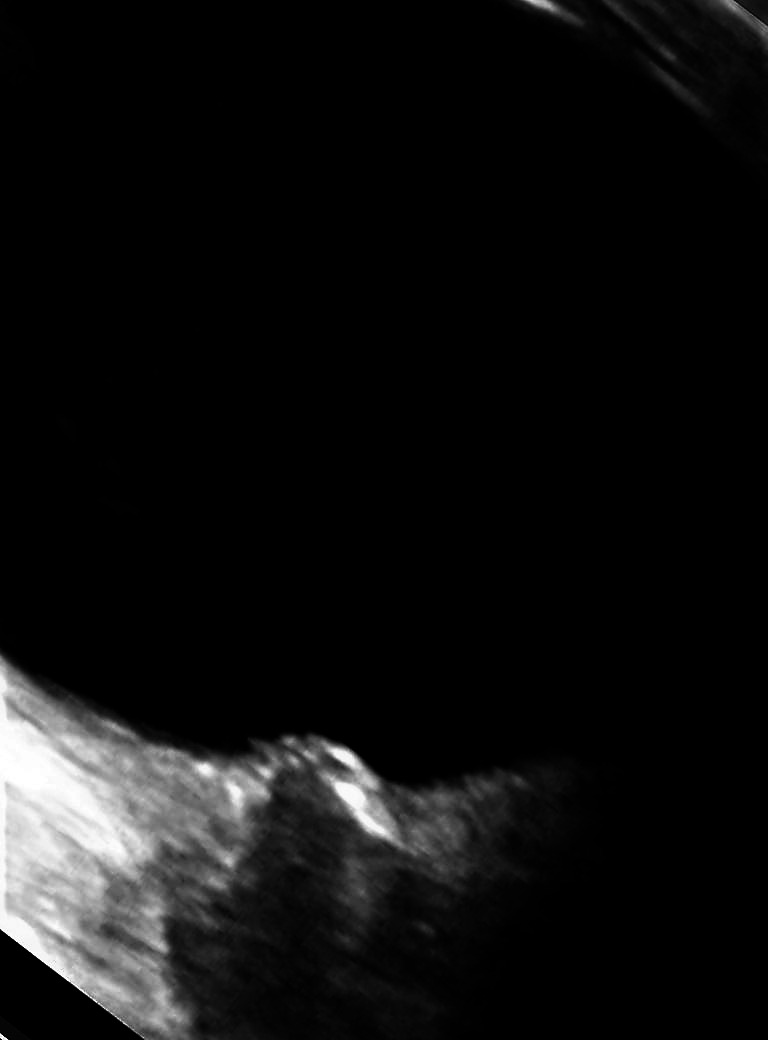
Right eye posterior structures, note the presence of increased optic nerve sheath diameter (ONSD) of 7mm (normal is up to 5mm) and the prominence of the optic nerve into the retina consistent with papilledema measuring 1.4mm from retina base.

**Figure 3:**
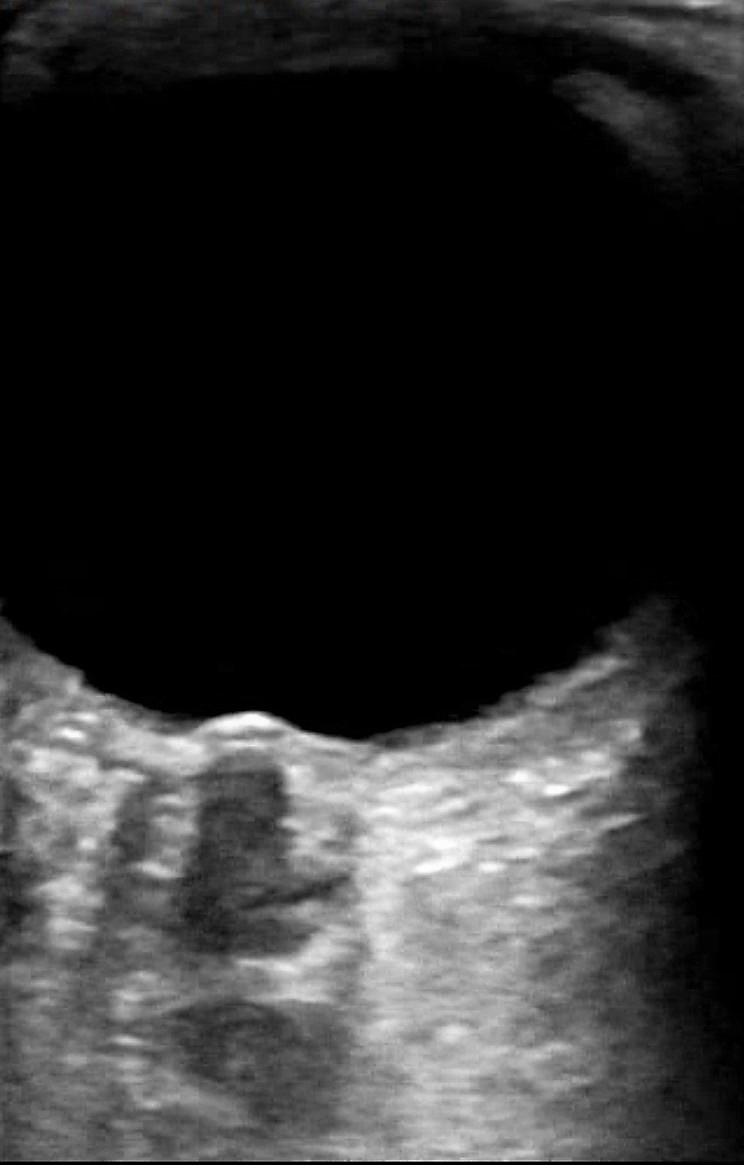
Left eye posterior structures, note the presence of increased optic nerve sheath diameter (ONSD) of 7.8mm (normal is up to 5mm), measuring 1.9mm from retina base. The altered probe position avoids the central mass to allow for accurate assessment.

The patient underwent ocular POCUS by a physician experienced in the modality. The patient was placed supine at a 45-degree bed angle. Given the report of left eye monocular diplopia, assessment began on the right eye which revealed optic nerve prominence, increased optic nerve sheath diameter (ONSD) consistent with papilledema (Figure 2 and Video S1). The quoted value of ONSD is taken from 3mm below the retinal insertion, using an upper limit of 5.0mm. The left globe also had papilledema with an ONSD of 7.8 mm (Figure 3)

**Figure 4:**
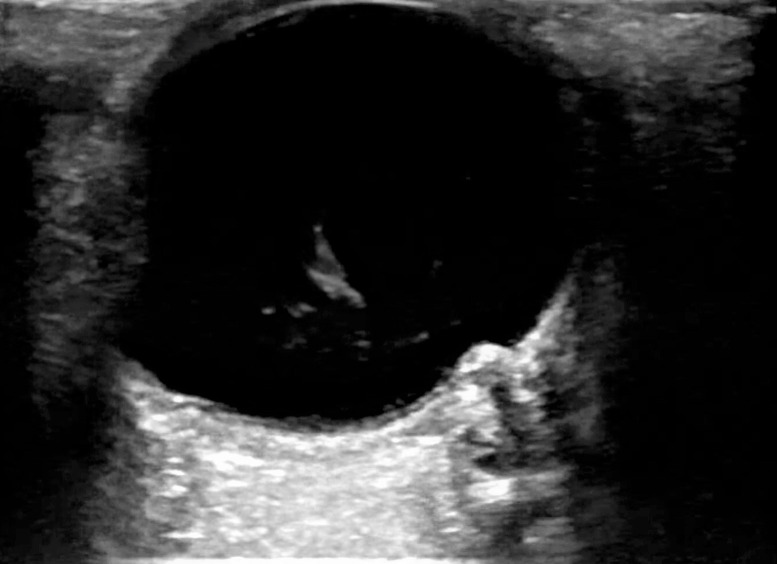
Left eye vitreous hemorrhage can now also be appreciated anterior to the prominent optic nerve.

On the assessment of the left eye, a dense irregular mass not fixed to the retina was also visualized (Figure 4). The dense intraorbital mass was seen rotating and the “washing machine sign” confirmed the lack of tethering of the mass to the retina through counter-rotation of the dense mass to the globe (Videos S2,S3). Given the presence of vitreous hemorrhage, a color waveform is applied to verify that central retinal arterial and venous flow is patent (Video S4). Given the patient’s positioning, the vitreous hemorrhage was seen to move inferioposteriorly from the probe with gravity. 

### Outcome

The ocular POCUS examination strongly supported the presence of intraocular hemorrhage within the left globe, which explained the patient’s monocular diplopia. Given the patient’s cerebral contusion and subarachnoid hemorrhage, the diagnosis of Terson syndrome was made.

The patient was urgently referred to ophthalmology for same-day review, who confirmed the presence of vitreous hemorrhage without complicating ocular features; the patient was referred for visual field testing. The patient remains under ophthalmology care and has an ongoing visual deficit. The current plan is for the patient to undergo regular visual field testing and close observation, aiming to avoid surgical intervention. Community neurorehabilitation continues as the patient suffers from a mild disability, personality changes, and chronic headaches. 

## Case Discussion

Terson syndrome is defined by the presence of vitreous or retinal hemorrhage (including pre-, intra-, and sub-retinal hemorrhage) in the context of intracranial hemorrhage, either arising from traumatic brain injury or subarachnoid hemorrhage. In the case of vitreous hemorrhage, this can be identified on ocular POCUS by the presence of a non-tethered dense mass within the globe. The gold standard for the diagnostic evaluation of Terson syndrome remains dilated ophthalmoscopy. However ocular POCUS has been found to have high sensitivity (80%) and specificity (100%) for the diagnosis of Terson syndrome 6. 

The Incidence of Terson syndrome remains unclear, with its presence potentially complicating more than 20% of all-cause subarachnoid hemorrhage (SAH) 7. Terson syndrome is likely under-reported outside of scientific study due to a lack of evaluation and awareness of the syndrome. As a result, patients with monocular Terson syndrome often face significant delay in referral to specialty care 8. It is important to note that delays in identifying Terson syndrome can result in poorer visual outcomes and associated morbidity. Patients with Terson syndrome who are not adequately managed are at risk of developing perimacular folds, retinal detachment, and ghost cell glaucoma 9.

The diagnosis of Terson syndrome can provide insight into the generalised clinical course in cases of intracranial hemorrhage. When grading head CTs using the Fisher scale, patients with low-grade SAH (grade I/II) are statistically unlikely to develop Terson syndrome when compared to those with high-grade SAH (grade III/IV) 10. The only independent predictor of Terson syndrome is raised intracranial pressure; however, the presence of Terson syndrome is associated with increasingly severe neurological impairment using Glasgow Coma Score and Hunt and Hess grading, higher mortality, and poorer overall neurological outcomes 11. It is important to note, however, that Terson syndrome may be present in patients with limited neurological compromise who make a full neurological recovery 12.

The mechanism by which Terson syndrome develops remains unclear, although as previously discussed, it is likely that raised intracranial pressure is important to its development 10. One proposed mechanism is that of glymphatic reflux, in which a reversal of the intraocular to subarachnoid glymphatic flow occurs. This would occur due to intraocular pressures suddenly being overcome by increases in intracranial pressure. In such a mechanism, intraocular blood products are derived directly from the subarachnoid hemorrhage 13. This mechanism may explain unilateral presentations of Terson syndrome given glymphatic flow is localised to each globe, particularly in cases where hemorrhage is limited to one cerebral hemisphere 14. Alternatively, optic nerve sheath compression due to raised intracranial pressure and increased central retinal vein pressure may lead to venous vascular dysfunction and associated hemorrhage 15. Alternative causes of acute raised intracranial pressure seldom propagate intraocular hemorrhage. Given this, it appears likely that the presence of intracranial hemorrhage, even in cases where hemorrhage is localised such as traumatic brain injury, is critical to the development of Terson syndrome.

In the case described above, ONSD was increased, but this was not isolated to the globe adjacent to the cerebral contusion. The role of ONSD generally, as well as its role in determining intracranial pressure, remains controversial. Given the determination of ONSD as a skill can be taught rapidly and in a reproducible manner, its use has expanded rapidly within the field of ocular POCUS 16. Smaller studies have shown ONSD to have a high correlation with raised intracranial pressure 17. To this end, meta-analysis has shown that although ONSD correlation with raised intracranial pressure is present, caution should still be taken in its use 18. Given how glymphatic reflux may play a fundamental role in the development of intraocular hemorrhage in Terson syndrome, ONSD may be less reliable in these patients. There has been no study to date exploring ONSD within the Terson syndrome cohort. 

## Conclusion

Terson syndrome is an important but challenging diagnosis in the acute care of patients suffering from intracranial injury. There are often significant delays in the diagnosis of Terson syndrome, leading to increased morbidity in patients requiring already requiring complex neurorehabilitation. This case illustrates that Ocular POCUS can be used to make a rapid diagnosis of Terson syndrome on the medical floor. 

## Disclosures

No conflicts of interest to declare, and all ethical guidance is followed where appropriate. 

## Patient Consent

Informed patient consent has been given to publish the respective imaging and information; all case data is anonymized in line with General Medical Council, local, and publisher guidance. 

## Supplementary Material

 Video S1 Right eye posterior structures. Note the presence of increased optic nerve sheath diameter (ONSD) and the prominence of the optic nerve into the retina consistent with papilledema.

Video S2 Left eye posterior structures with vitreous hemorrhage appreciated moving in an anti-clockwise direction anterior to the prominent optic nerve.

Video S3 Left eye when the patient is asked to look left and right; this leads to rotation on the eyeball and counter-rotation of the mass; this pathological sign is known as the ‘washing machine sign’ and is consistent with vitreous hemorrhage.

Video S4 Left eye with pulsatile retinal artery flow appreciated alongside concomitant flow from the central retinal vein excluding acute vascular compromise of the affected eye.
